# Tumor Microenvironment-Responsive Nanomaterials as Targeted Delivery Carriers for Photodynamic Anticancer Therapy

**DOI:** 10.3389/fchem.2020.00758

**Published:** 2020-09-29

**Authors:** Houhe Liu, Jiwen Yao, Huanhuan Guo, Xiaowen Cai, Yuan Jiang, Mei Lin, Xuejun Jiang, Wingnang Leung, Chuanshan Xu

**Affiliations:** ^1^Key Laboratory of Molecular Target and Clinical Pharmacology, State Key Laboratory of Respiratory Disease, School of Pharmaceutical Science & Fifth Affiliated Hospital, Guangzhou Medical University, Guangzhou, China; ^2^Asia-Pacific Institute of Aging Studies, Lingnan University, Hong Kong, China

**Keywords:** photodynamic therapy, photosensitizer, drug delivery, tumor microenvironment, stimuli-responsive nanomaterials

## Abstract

Photodynamic therapy (PDT), as an alternative approach to treat tumors through reactive oxygen species (ROS) produced by the activated photosensitizers (PS) upon light irradiation, has attracted wide attention in recent years due to its low invasive and highly efficient features. However, the low hydrophilicity and poor targeting of PS limits the clinical application of PDT. Stimuli-responsive nanomaterials represent a major class of remarkable functional nanocarriers for drug delivery. In particular, tumor microenvironment-responsive nanomaterials (TMRNs) can respond to the special pathological microenvironment in tumor tissues to release the loaded drugs, that allows them to control the release of PS within tumor tissues. Recent studies have demonstrated that TMRNs can achieve the targeted release of PS at tumor sites, increase the concentration of PS in tumor tissues, and reduce side effects of PDT. Hence, in the present paper, we review TMRNs, mainly including pH-, redox-, enzymes-, and hypoxia-responsive smart nanomaterials, and focus on the application of these smart nanomaterials as targeted delivery carriers of PS in photodynamic anticancer therapy, to further boost the development of PDT in tumor therapy.

## Introduction

Photodynamic therapy (PDT) is a promising approach to treat malignancies and other non-neoplastic lesions including condyloma acuminata, acne, and port wine stains (Rkein and Ozog, 2014). For decades, safe and effective PDT in the management of cancer has attracted extensive attention in clinical settings (Kelly et al., 1975; Chang et al., 2018; Feng et al., 2018; Sun et al., 2019). However, poor targeting and low solubility of most photosensitizers (PS) limits the clinical application of PDT (Panagopoulos et al., 1989; Haddad et al., 2000; Dolmans et al., 2003; Chatterjee et al., 2008). Recently, nanomaterials have shown great promise for improving the solubility and targeting of PS (Lieber, 2003; Roco, 2003a,b). Nanoformulations can not only reduce the side effects of PS, but also increase the therapeutic effect of PDT through controlling the delivery of PS in the tumor tissues. However, it is very difficult for conventional nanomaterials such as liposomes, micelles, dendrimers, and polymeric nanoparticles to deliver PS precisely to tumor lesions via the enhanced permeability and retention effect (EPR) (Marcucci et al., 2016). It is well-known that tumor tissues are a complex system consisting of tumor cells and their surrounding cellular and extracellular materials. The tumor microenvironment (TME) is composed of tumor cells and tumor stroma (Ramamonjisoa and Ackerstaff, 2017). In TME, there are diverse cell types including fibroblasts, pericytes, endothelial cells, dendritic cells, smooth muscle cells, inflammatory cells, and cancer stem cells (CSCs). The TME-forming cells interact with tumor cells to create a unique pathological TME over the normal tissues, including hypoxia, low pH, overexpressed enzymes, and redox conditions (Liu and Huskens, 2015; Tian et al., 2017). The unique features of TME motivate many researchers to develop tumor microenvironment-responsive nanomaterials (TMRNs) as drug carriers for precisely delivering the loaded drugs to enhance drug concentrations in tumor cells through responding to the specific pathological microenvironment in tumor tissues (Muthu et al., 2009; Wei et al., 2013; Karimi, 2015; Paris et al., 2015; Nazemi et al., 2016). Most recently, TMRNs as delivery carriers of PS have been widely researched and developed in PDT on tumors. In the present article, we focus on reviewing the application of TMRNs as targeted delivery carriers for photodynamic anticancer therapy, including its principle and defect and update new research progress to enrich and promote the development of PDT.

## Principle of PDT

PDT is based on a reactive oxygen species (ROS) generated from light-activated PS to kill cancer cells (Pervaiz and Olivo, 2006; Li et al., 2019). When PS is irradiated by light at a specific wavelength, the excited PS transfers from a single-electron state to a low-lying or high-lying electronic singlet state, and then reaches the excited triplet state by intersystem crossing. Triplet PS becomes a ground state by collision with ground-state triplet molecular oxygen, and triplet molecular oxygen is excited into a singlet electronically excited state, which in turn produces singlet oxygen and other ROS, including superoxide anions (${\text{O}}_{2}^{-}$), hydroxyl radicals, and hydrogen peroxide (H_2_O_2_). These ROS can damage most types of biomolecules. ROS are the direct effectors that PDT kills tumor cells with (Oleinick et al., 2002; Jain et al., 2017; Dobson et al., 2018; Jiang et al., 2019), however, most PS lack targeting capability and induce cytotoxicity on neighbor normal cells by releasing ROS during photodynamic anticancer therapy. Thus, there is an urgent need to precisely control the production of ROS in target cells for improving the clinical outcome of PDT.

## TMRNs as Targeted Delivery Carriers for Photodynamic Anticancer Therapy

**Figure 1:**
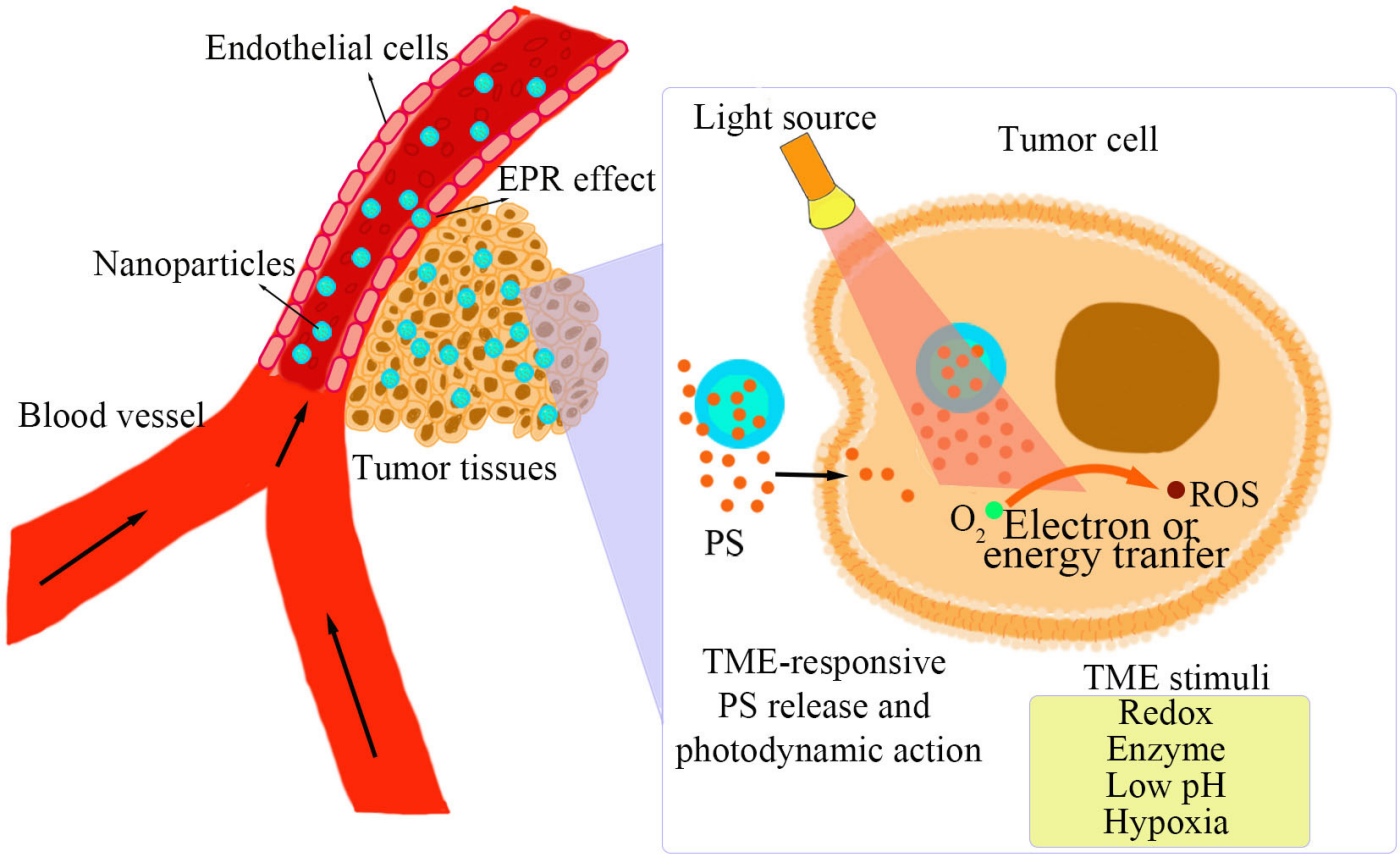
TME-responsive PS release and photodynamic action.

A TME has unique pathological conditions over normal tissues, including low pH, high GSH, hypoxia, and some specific enzymes highly expressed in tumor tissues (Upreti et al., 2013). On the basis of the unique features in TME, TMRNs have been developed as a novel smart nanoplatform that can intelligently respond to special pathological conditions in TME, such as pH-responsive, redox-responsive, hypoxia-responsive, enzyme-responsive, and multiple stimuli-responsive nanomaterials, for specifically delivering PS to tumor tissues (Zhu et al., 2017). As shown in Figure 1, after the TMRNs reach the tumor site and enter the tumor tissue due to the EPR effect. Then, the photosensitizer is released inside or outside the tumor cells in response to the TME stimuli. When the photosensitizer is irradiated by excitation light, ROS will be generated by electron transfer or energy transfer. The detailed information of the reaction principles and reaction sites of several stimulus response nanomaterials mentioned below are shown in Table 1.

**Table 1:** Summary of tumor microenvironment-responsive nanomaterials for PDT.

**Types of stimuli-responsive nanomaterials**	**Nanomaterials**	**Photosensitizer**	**Responsive site**	**Principle of response**	**Application**	**References**
pH-responsive	DEAK-DMA	Protoporphyrin IX	2,3-dimethylmaleic anhydride	Amide bond breaks	PDT for hela cells and H22 tumors	Han et al., 2017
	DAA	Diketopyrrolopyrrole	Diethylaminop-henyl	The protonation of Diethylaminop-henyl	PDT for hela cells / PDT for hela tumors	Liang et al., 2018
	HSA–Ce6/TAM	Ce6	Tamoxifen	The protonation of tamoxifen	PDT for 4T1 cells / PDT for 4T1 tumors	Yang et al., 2018
	PEG-b-cPPT	2-(1-hexyloxyethyl)-2-devinyl pyropheophorbide-a	Tertiary amines	The protonation of tertiary amines	PDT for B16F10 cells and MC38 tumors	Yang et al., 2020
Redox-responsive	Ds-sP/TCPP-T^ER^	4,4′,4′′,4′′′-(porphyrin-5,10,15,20-tetrayl)tetrakis(N-(2-((4-methylphenyl) sulfonamido)ethyl)benzamide	Disulfide bond	GSH causes Disulfide reduction	PDT for 4T1, MDA-MB-435, MDA-MB-231 and MCF-7 cells / PDT for 4T1 tumors	Deng et al., 2020
	DPPSC	Protoporphyrin IX	Disulfide bond	GSH causes Disulfide reduction	PDT for PANC-1 cells / PDT for PANC-1 tumor	Wang et al., 2017
Hypoxia-responsive	HCHOA	Ce6	Azobenzene group	Azobenzene derivatives reduce to aniline derivatives	PDT for 4T1 cells / PDT for 4T1 tumors	Yang G. et al., 2019
	TENAB	ENAB	TPZ	Hypoxia-responsive to produce (OH^•^)	PDT for hela cells / PDT for hela tumors	Chen et al., 2019
	PA/HA-Ce6@TPZ	Ce6	Alkylated 2-nitroimidazole	Alkylated 2-nitroimidazole reduced to alkyl-2-aminoimidazole	PDT for 4T1 and L929 cells/PDT for 4T1 tumors	Zhu et al., 2019
Enzyme-responsive	DBHA	Diiodo-styryl-BODIPY	HA	Hyaluronidase causes HA degradation	PDT for HCT-116 cells/PDT for HCT-116 tumors	Shi et al., 2016
	BCPs	coumarin and Nile blue	Quinone trimethyl	NQO1 enzyme triggers self-immolative cleavage of quinone	PDT for A549 cells/PDT for A549 tumors	Yao et al., 2020
Multiple stimuli-responsive	Ce6-Ns	Ce6	HSA and disulfide bond	Proteases causes HSA dissociate. Low pH reduced the electrostatic adsorption of HAS. GSH causes Disulfide reduction	PDT for HeLa, B16, and MCF-7 cells / PDT for MCF-7 tumors	Zhang et al., 2016
	pSiO_2_ -ss-HA/CHI	Carbon quantum dots	Disulfide bonds, hydrogen bonds and HA	Hyaluronidase causes HA dissociate. Low pH causes swelling or shedding of the HA/CHI layer. GSH causes Disulfide reduction	PDT for HCT-116 cells	Chen et al., 2020

### pH-Responsive Nanomaterials

Under normal circumstances, the pH of extracellular tissues and blood is usually maintained at around 7.4. Due to the high rate of glycolysis, the pH in solid tumors reduces to around 5.0–6.8 (Park et al., 2007; Chen et al., 2016; Liu et al., 2016). In addition, the pH in lysosomes is also lower than other organelles in tumor cells. Therefore, pH can be widely used as a stimuli approach for the precise delivery of PS. Currently, the design strategy for pH-responsive nanomaterials used in PDT is mainly based on chemical bond breaking in the low pH environment. Han et al. (2017) found shape-switched tumor extracellular pH-responsive chimeric peptide (named DEAK-DMA)-based nanoparticles to enhance tumor uptake of PS on the basis of acidic condition-induced detachment of the dimethylmaleic anhydride group. DEAK-DMA could self-assemble into spherical nanoparticles under physiological conditions. In the acidic microenvironment in tumor tissues, DEAK-DMA undergoes disruption of the acid-sensitive 2,3-dimethylmaleic anhydride group. Then the restoration of ionic complementarity between the DEAK-DMA induced the formation of rod-shaped nanoparticles, thereby enhancing uptake of PS in the tumor cells. The protonation of certain groups in an acidic environment can also affect the physical and chemical properties of the entire molecule (Yang X. D. et al., 2019), which also provides an alternative strategy for designing pH-responsive nanomaterials. Liang et al. (2018) designed a biocompatible pH-responsive nanoparticle (named DAA) by the self-assembly approach to exhibit effective PDT/photothermal anticancer activities due to the protonation of diethylaminophenyl. In addition, the anti-vascular drug 5,6-dimethylxanthine-4-acetic acid was combined in the DAA nanoparticles for targeting the vascular endothelial growth factor and was found to release from the weakly acidic endocytic organelles of endothelial cells by hydrolysis of the ester bond, and effectively prevent the spread and metastasis of tumors. Yang et al. (2018) used tamoxifen to fabricate a pH-responsive nanoparticle (named HSA–Ce6/TAM) for PDT via the self-assembly of the human serum albumin (HSA) modified with Chlorin e6 (Ce6). The protonated tamoxifen dissociated it from HSA–Ce6/TAM in the acidic condition to push the HSA–Ce6/TAM nanoparticles (≈130 nm) to break down into smaller nanoparticles (≈10 nm), which promoted Ce6 uptake into target cells.

*In situ* burst releases of tumor antigens induced by PDT significantly initiated the immune response (Ng et al., 2018; Meng et al., 2019a; Wang et al., 2019). Yang et al. (2020) prepared the pH-responsive double load nanovesicles (named PEG-b-cPPT) by self-assembly of block copolymer polyethylene glycol-b-cationic polypeptide. In the acidic environment, the double-loaded nanoparticles released the PS and indoximod into the cytoplasm because of the protonation of the tertiary amine in the cationic polypeptide. The nanovesicles were not only the carriers of PS, but also induced immunogenic cell death upon light irradiation, providing a novel strategy for the combination of PDT and immunotherapy.

### Redox-Responsive Nanomaterials

Redox-responsive nanomaterials can effectively deliver and release loaded drugs to target cells because the glutathione (GSH) concentration in tumor tissues is four times that in normal tissues (Mo and Gu, 2016). In addition, the intracellular GSH level is usually higher compared with the extracellular environment. Thus, redox-responsive nanomaterials are also expected to be used for targeted delivery (Fukino et al., 2017; Raza et al., 2018). The disulfide bonds are converted into sulfhydryl groups after being reduced by the action of GSH, which leads to the destruction of the nanoparticles. Meanwhile, due to the stability of the disulfide bonds in the external environment, the redox-responsive nanomaterials can protect the loaded drugs from premature release (Iqbal and Keshavarz, 2018). Deng et al. (2020) synthesized a nanoparticle (named Ds-sP/TCPP-T^ER^) containing disulfide reduction-sensitizer and an endoplasmic reticulum targeting PS, which could induce endoplasmic reticulum stress through ROS in the endoplasmic reticulum and enhance immunogenic cell death to activate immune activity. Wang et al. (2017) constructed a powerful and intelligent “all in one” protoporphyrin-based polymer nanoplatform (named DPPSC) that had the ability to enhance chemotherapy-PDT by gradually and intelligently responding to low pH in lysosomes and high concentration GSH in cytoplasm. The polymer consisted of dextran grafted by protoporphyrin IX as a hydrophilic segment and the anticancer drug camptothecin was coupled to dextran through a disulfide bond containing a pH-sensitive linker as a hydrophobic segment. The use of photochemical internalization enhanced nanoparticles in tumor cells and subsequently released PS and camptothecin through pH and GSH responses, achieving the synergistic therapy of PDT and chemotherapeutic drugs.

### Hypoxia-Responsive Nanomaterials

Hypoxia is the most common phenomenon in a majority of solid tumors, which provides opportunities for tumor-specific diagnosis and treatment triggered by hypoxia-responsive nanomaterials (Vordermark, 2010; McKeown, 2014). Growing evidence shows that azobenzene derivatives can be reduced to aniline derivatives by various reductases in the hypoxia environment (Mirabello et al., 2018). Owing to excellent hypoxia sensitivity, azobenzene derivatives have widely been used to detect hypoxia levels as hypoxia-reactive fluorescent probes as well as deliver drug or genes to hypoxic cancer cells for anticancer therapy (Perche et al., 2014; Dong et al., 2020). Yang G. et al. (2019) cross-linked the hypoxia-sensitive azophenyl group between the HSA coupled with the Ce6 and the HSA combined with the oxaliplatin prodrug to prepare a unique hypoxia-responsive nanosystem (named HCHOA). HCHOA was stable under normal tissue. In a hypoxic tumor, the azobenzene group in HCHOA nanoparticles will be cleaved by reductase and dissociated into small particle size complexes (Ho and HC) with diameters of <10 nm, which can significantly improve the penetration ability of the nanoparticles and enhance the accumulation of photosensitizers and drugs in tumor tissues. Tirapazamine (TPZ) is a promising hypoxic-specific prodrug that can be activated under hypoxic conditions to produce hydroxyl radicals (HO^•^). Chen et al. (2019) encapsulated TPZ and aza-BODIPY derivatives in eutectic materials by the use of oleic acid and linoleic acid to prepare a near infrared activated and hypoxia-responsive nanomaterial (named TENAB) for the combined treatment of PDT and chemotherapy. The release of TPZ is triggered by the hyperthermia generated by aza-BODIPY derivatives under laser irradiation at 808 nm. Meanwhile, when activated at pH 5.0, aza-BODIPY derivatives in molten TENAB NPs will switch the charge transfer (CT) state to produce ROS and consume oxygen to aggravate the hypoxic environment. TPZ was reduced to its cytotoxic form, producing hydroxyl radical (HO^•^) to enhance PDT efficiency. Zhu et al. (2019) designed tumor-targeted, low-oxygen dissociable nanoparticles (named PA/HA-Ce6@TPZ) for the delivery of Ce6 and low-oxygen activating drug TPZ. After irradiation with Ce6 light, tumor cells enter endocytosis and produce high concentration of ROS, which leads to apoptosis and a local hypoxia environment.

### Enzyme-Responsive Nanomaterials

Compared with normal tissues, certain enzymes such as matrix metalloproteinase, hyaluronidase, β-glucuronidase, and esterase are usually overexpressed in the tumor microenvironment (Lopez-Otin and Bond, 2008; McAtee et al., 2014). The selectivity and effectivity of enzymatic reactions endow enzyme-responsive nanomaterials with an extensive prospect in the targeted delivery and precise release of PS. Hyaluronic acid (HA) is a negatively charged natural glycosaminoglycan, widely distributed in the human body. HA has good biocompatibility and can target the CD44 receptor overexpressed in many types of cancer cells (Toole, 1990). When reaching the tumor tissues, hyaluronidase within tumor tissues can degrade HA (Choi et al., 2019). Shi et al. (2016) used diiodo-styryl-BODIPY as a PS, and then conjugated HA to prepare hyaluronidas-responsive nanoparticles (named DBHA) as activatable photodynamic theranostics for treating cancer. In normal tissues, because of the aggregation of diiodo-styryl-BODIPY in DBHA, they limit the production of ROS. However, after endocytosis of tumor cells, HA of DBHA was degraded by hyaluronidase in lysosome, and then diiodo-styryl-BODIPY was released and induced the PDT activity in tumor cells.

NADPH: quinone oxidoreductase isoenzyme 1 (NQO1) is a very special enzyme, which can catalyze the two-electron reduction of quinone (Oh and Park, 2015). Numerous studies have shown that NQO1 is upregulated in breast cancer, pancreatic cancer, colorectal cancer, cervical cancer, and lung cancer (Ma et al., 2014; Yang et al., 2014). Yao et al. (2020) reported that NQO1-responsive multifunctional polymer vesicles (named BCPs) covalently conjugated with PS (coumarin and Nile Blue). In the absence of NQO1, due to the “dual quenching” effect, that is to say, the quenching caused by aggregation caused by photoinduced electron transfer (PET) and the quenching of quinone production, the fluorescence emission and PDT efficiency were in the “off” state. After the NQO1-responsive nanovesicles entered into the tumor cells, the NQO1 in the tumor cells triggered the self-immolative cleavage of the quinone trimethyl lock, the release of the PS, and the simultaneous NIR emission and PDT activation.

### Multiple Stimuli-Responsive Nanomaterials

Due to the unique characteristics of the tumor microenvironment, stimuli-responsive nanomaterials are extensively accepted as targeted delivery and precise release carriers in photodynamic anticancer therapy (Yang et al., 2018; Ma et al., 2019). Many new advances have been made in developing stimuli-responsive nanomaterials in PDT, however, most stimuli-responsive nanomaterials are only responsive to a single stimulation (Zhang et al., 2017). Tumor tissues are actually very complex biological systems with low pH, hypoxia, redox, and enzymes overexpressed in the microenvironment (Klaikherd et al., 2009). Multiple stimuli-responsive nanomaterials could respond to two or more types of stimuli in the tumor environment simultaneously, which show great promise in more precise delivery and release of drugs to target sites (Zhang et al., 2016). Zhang et al. (2016) used HSA and poly-l-lysine with surface modification by polyethylene glycol to design and prepare a multiple stimuli-responsive nanomaterial (named Ce6-Ns) according to an electrostatic assembly strategy. Then Ce6, protoporphyrin IX, or verteporfin were loaded in the nanomaterials to prepare a pH/redox/enzyme-responsive protein nanospheres for photodynamic tumor ablation. When the nanoparticles reached the tumor tissues, the proteases overexpressed by the tumor cells decomposed HSA and caused the HSA to dissociate. Under the influence of the HSA isoelectric point, the acidic condition in the tumor tissues reduced the electrostatic adsorption of HSA, and the disulfide bonds in HSA were reduced by the overexpressed GSH in tumor cells. Therefore, Ce6-Ns can effectively enhance the accumulation of Ce6 in tumor sites and improve the efficiency of PDT. Chen et al. (2020) prepared a triplet responsive porous silica carrier (named pSiO2 -ss-HA/CHI) to load carbon quantum dots and doxorubicin for photodynamic / chemotherapy. Firstly, an amino-functional porous silica nanoparticles with central radial pores were prepared using an emulsion method, and then succinic acid and cystamine were successively grafted onto the surface of the nanoparticles via amide bonds as linkers. Subsequently, doxorubicin and carbon quantum dots were loaded in the nanoparticles. Finally, the surface of the carrier was coated with HA and chitosan to block the drug-loading holes. Due to the presence of disulfide bonds, amino bonds, and hydrogen bonds in the nanoparticles, the nanomaterials showed pH-, redox-, and enzyme-responsive features.

## Summary and Outlook

With the development of laser medicine and material science in recent years, PDT has become a promising treatment for combating malignancies. However, poor targeting and low water solubility of the conventional PS limit the application of PDT in clinical settings. TMRNs, including pH-, redox-, hypoxia-, and enzyme-responsive nanoparticles, have been proposed as targeted delivery carriers of PS to enhance the therapeutic efficacy of PDT. Moreover, a tumor is a complex and refractory disease. Single therapy is often difficult to cure it. Combined therapy has become a main strategy in the management of malignancies using multiple approaches such as chemotherapy, immunotherapy, and PDT (Liu et al., 2017; Cheng et al., 2019; Meng et al., 2019b). TMRNs as a smart delivery carrier could be a favorable “bridge” to load PS, chemotherapeutic drugs, and immune-enhancing drugs together to precisely deliver and release multiple drugs to target cells, achieving synergistic treatment of PDT, chemotherapy, and immunotherapy. As of now TMRNs have been confirmed as targeted delivery carriers of PS for PDT in *in-vitro* and *in-vivo* models. However, few clinical trials have investigated the precise delivery of PS for PDT on tumors using TMRNs. A main challenge is the complexity of TMRNs such as tedious preparation, complicated characterization, and uncertainty of the *in-vivo* fate of TMRNs. Thus, addressing these shortcomings should be an important task for translating TMRNs as a targeted delivery carrier of PS for the clinical application of PDT.

## Author Contributions

HL: principal writer. CX and WL: article revision and review before submission. JY, HG, XC, YJ, ML, and XJ: provide written suggestions. All authors contributed to the article and approved the submitted version.

## Conflict of Interest

The authors declare that the research was conducted in the absence of any commercial or financial relationships that could be construed as a potential conflict of interest.

## References

[B1] Chang Y. Cheng Y. Feng Y. Jian H. Wang L. Ma X.. (2018). Resonance energy transfer-promoted photothermal and photodynamic performance of Gold-copper sulfide yolk-shell nanoparticles for chemophototherapy of cancer. Nano. Lett. 18, 886–897. 10.1021/acs.nanolett.7b04162 29323915

[B2] Chatterjee D. K. Fong L. S. Zhang Y. (2008). Nanoparticles in photodynamic therapy: An emerging paradigm. Adv. Drug. Deliver. Rev. 60, 1627–1637. 10.1016/j.addr.2008.08.003 18930086

[B3] Chen D. Tang Y. Zhu J. Zhang J. Song X. Wang W.. (2019). Photothermal-pH-hypoxia responsive multifunctional nanoplatform for cancer photo-chemo therapy with negligible skin phototoxicity. Biomaterials 221:119422. 10.1016/j.biomaterials.2019.119422 31437723

[B4] Chen Q. Liu X. Zeng J. Cheng Z. Liu Z. (2016). Albumin-NIR dye self-assembled nanoparticles for photoacoustic pH imaging and pH-responsive photothermal therapy effective for large tumors. Biomaterials 98, 23–30. 10.1016/j.biomaterials.2016.04.041 27177219

[B5] Chen Y. Li X. Zhao Y. Zhang X. Sun L. (2020). Preparation of triple-responsive porous silica carriers and carbon quantum dots for photodynamic-/chemotherapy and multicolor cell imaging. Chem. Nano. Mat. 6, 648–656. 10.1002/cnma.201900777

[B6] Cheng H. Fan G. L. Fan J. H. Zheng R. R. Zhao L. P. Yuan P.. (2019). A self-delivery chimeric peptide for photodynamic therapy amplified immunotherapy. Macromol. Biosci. 19:e1800410. 10.1002/mabi.201800410 30576082

[B7] Choi K. Y. Han H. S. Lee E. S. Shin J. M. Almquist B. D. Lee D. S.. (2019). Hyaluronic acid–based activatable nanomaterials for stimuli-responsive imaging and therapeutics: beyond CD44-mediated drug delivery. Adv. Mater. 31:e1803549. 10.1002/adma.201803549 30773699

[B8] Deng H. Zhou Z. Yang W. Lin L. Wang S. Niu G.. (2020). Endoplasmic reticulum targeting to amplify immunogenic cell death for cancer immunotherapy. Nano. Lett. 20, 1928–1933. 10.1021/acs.nanolett.9b05210 32073871

[B9] Dobson J. de Queiroz G. F. Golding J. P. (2018). Photodynamic therapy and diagnosis: Principles and comparative aspects. Vet. J. 233, 8–18. 10.1016/j.tvjl.2017.11.012 29486883

[B10] Dolmans D. E. Fukumura D. Jain R. K. (2003). Photodynamic therapy for cancer. Nat. Rev. Cancer 3, 380–387. 10.1038/nrc1071 12724736

[B11] Dong X. Mu L. L. Liu X. L. Zhu H. Yang S. C. Lai X. (2020). Biomimetic, hypoxia-responsive nanoparticles overcome residual chemoresistant leukemic cells with Co-targeting of therapy-induced bone marrow niches. Adv. Funct. Mater. 30:2000309 10.1002/adfm.202000309

[B12] Feng L. Gai S. Dai Y. He F. Sun C. Yang P. (2018). Controllable generation of free radicals from multifunctional heat-responsive nanoplatform for targeted cancer therapy. Chem. Mater. 30, 526–539. 10.1021/acs.chemmater.7b04841

[B13] Fukino T. Yamagishi H. Aida T. (2017). Redox-responsive molecular systems and materials. Adv. Mater. 29:1603888. 10.1002/adma.201603888 27990693

[B14] Haddad R. Kaplan O. Greenberg R. Siegal A. Skornick Y. Kashtan H. (2000). Photodynamic therapy of murine colon cancer and melanoma using systemic aminolevulinic acid as a photosensitizer. Int. J. Surg. Investig. 2, 171–178. 12678516

[B15] Han K. Zhang J. Zhang W. Wang S. Xu L. Zhang C.. (2017). Tumor-triggered geometrical shape switch of chimeric peptide for enhancedin vivo tumor internalization and photodynamic therapy. ACS. Nano. 11, 3178–3188. 10.1021/acsnano.7b00216 28296387

[B16] Iqbal H. M. N. Keshavarz T. (2018). “14 - Bioinspired polymeric carriers for drug delivery applications,” in Stimuli Responsive Polymeric Nanocarriers for Drug Delivery Applications, Volume 1, eds. Makhlouf A.S.H. N.Y. (Abu-Thabit: Woodhead Publishing), 377–404. 10.1016/B978-0-08-101997-9.00018-7

[B17] Jain M. Zellweger M. Wagnières G. van den Bergh H. Cook S. Giraud M. (2017). Photodynamic therapy for the treatment of atherosclerotic plaque: Lost in translation? Cardiovasc. Ther. 35:e12238. 10.1111/1755-5922.12238 27893195

[B18] Jiang Y. Xu C. Leung W. Lin M. Cai X. Guo H.. (2019). Role of exosomes in photodynamic anticancer therapy. Curr. Med. Chem. 10.2174/0929867326666190918122221. [Epub ahead of print]. 31533597

[B19] Karimi M. (2015). Smart Internal Stimulus-Responsive Nanocarriers for Drug and Gene Delivery. San Rafael California (40 Oak Drive, San Rafael, CA, 94903, USA); Bristol England (Temple Circus, Temple Way, Bristol BS1 6HG, UK): Morgan & Claypool Publishers. 10.1088/978-1-6817-4257-1

[B20] Kelly J. F. Snell M. E. Berenbaum M. C. (1975). Photodynamic destruction of human bladder carcinoma. Br. J. Cancer 31, 237–244. 10.1038/bjc.1975.30 1164470 PMC2009375

[B21] Klaikherd A. Nagamani C. Thayumanavan S. (2009). Multi-stimuli sensitive amphiphilic block copolymer assemblies. J. Am. Chem. Soc. 131, 4830–4838. 10.1021/ja809475a 19290632 PMC2693022

[B22] Li X. Zheng B. Peng X. Li S. Ying J. Zhao Y. (2019). Phthalocyanines as medicinal photosensitizers: Developments in the last five years. Coordin. Chem. Rev. 379, 147–160. 10.1016/j.ccr.2017.08.003

[B23] Liang P. Huang X. Wang Y. Chen D. Ou C. Zhang Q.. (2018). Tumor– microenvironment-responsive nanoconjugate for synergistic antivascular activity and phototherapy. ACS. Nano. 12, 11446–11457. 10.1021/acsnano.8b06478 30345740

[B24] Lieber C. M. (2003). Nanoscale science and technology: Building a big future from small things. MRS. Bull. 28, 486–491. 10.1557/mrs2003.144

[B25] Liu J. Huskens J. (2015). Bi-compartmental responsive polymer particles. Chem. Commun. (Camb). 51, 2694–2697. 10.1039/C4CC08413F 25574761

[B26] Liu L. Fu L. Jing T. Ruan Z. Yan L. (2016). pH-triggered polypeptides nanoparticles for efficient BODIPY imaging-guided near infrared photodynamic therapy. ACS. Appl. Mater. Interfaces 8, 8980–8990. 10.1021/acsami.6b01320 27020730

[B27] Liu W. Wang Y. M. Li Y. H. Cai S. J. Yin X. B. He X. W.. (2017). Fluorescent imaging-guided chemotherapy-and-photodynamic dual therapy with nanoscale porphyrin metal-organic framework. Small 13:1603459. 10.1002/smll.201603459 28244202

[B28] Lopez-Otin C. Bond J. S. (2008). Proteases: multifunctional enzymes in life and disease. J. Biol. Chem. 283, 30433–30437. 10.1074/jbc.R800035200 18650443 PMC2576539

[B29] Ma Y. Kong J. Yan G. Ren X. Jin D. Jin T.. (2014). NQO1 overexpression is associated with poor prognosis in squamous cell carcinoma of the uterine cervix. BMC. Cancer 14:414. 10.1186/1471-2407-14-414 24912939 PMC4058702

[B30] Ma Z. Hu P. Guo C. Wang D. Zhang X. Chen M.. (2019). Folate-mediated and pH-responsive chidamide-bound micelles encapsulating photosensitizers for tumor-targeting photodynamic therapy. Int. J. Nanomed. 14, 5527–5540. 10.2147/IJN.S208649 31413561 PMC6661377

[B31] Marcucci F. Stassi G. De Maria R. (2016). Epithelial-mesenchymal transition: a new target in anticancer drug discovery. Nat. Rev. Drug. Discov. 15, 311–325. 10.1038/nrd.2015.13 26822829

[B32] McAtee C. O. Barycki J. J. Simpson M. A. (2014). Emerging roles for hyaluronidase in cancer metastasis and therapy. Adv. Cancer. Res. 123, 1–34. 10.1016/B978-0-12-800092-2.00001-0 25081524 PMC4445717

[B33] McKeown S. R. (2014). Defining normoxia, physoxia and hypoxia in tumours-implications for treatment response. Br. J. Radiol. 87:20130676. 10.1259/bjr.20130676 24588669 PMC4064601

[B34] Meng Z. Zhou X. Xu J. Han X. Dong Z. Wang H.. (2019a). Light-triggered in situ gelation to enable robust photodynamic-immunotherapy by repeated stimulations. Adv. Mater. 31:e1900927. 10.1002/adma.201900927 31012164

[B35] Meng Z. Zhou X. Xu J. Han X. Dong Z. Wang H.. (2019b). Light-triggered in situ gelation to enable robust photodynamic-immunotherapy by repeated stimulations. Adv. Mater. 31:e1900927. 31012164 10.1002/adma.201900927

[B36] Mirabello V. Cortezon-Tamarit F. Pascu S. I. (2018). Oxygen sensing, hypoxia tracing and in vivo imaging with functional metalloprobes for the early detection of non-communicable diseases. Front. Chem. 6:27 10.3389/fchem.2018.00027 29527524 PMC5829448

[B37] Mo R. Gu Z. (2016). Tumor microenvironment and intracellular signal-activated nanomaterials for anticancer drug delivery. Mater. Today 19, 274–283. 10.1016/j.mattod.2015.11.025

[B38] Muthu M. S. Rajesh C. V. Mishra A. Singh S. (2009). Stimulus-responsive targeted nanomicelles for effective cancer therapy. Nanomedicine 4, 657–667. 10.2217/nnm.09.44 19663594

[B39] Nazemi A. Boott C. E. Lunn D. J. Gwyther J. Hayward D. W. Richardson R. M.. (2016). Monodisperse cylindrical micelles and block comicelles of controlled length in aqueous media. J. Am. Chem. Soc. 138, 4484–4493. 10.1021/jacs.5b13416 27049840

[B40] Ng C. W. Li J. Pu K. (2018). Recent progresses in phototherapy-synergized cancer immunotherapy. Adv. Funct. Mater. 28:1804688 10.1002/adfm.201804688

[B41] Oh E. T. Park H. J. (2015). Implications of NQO1 in cancer therapy. BMB. Rep. 48, 609–617. 10.5483/BMBRep.2015.48.11.190 26424559 PMC4911202

[B42] Oleinick N. L. Morris R. L. Belichenko I. (2002). The role of apoptosis in response to photodynamic therapy: what, where, why, and how. Photoch. Photobio. Sci. 1, 1–21. 10.1039/b108586g 12659143

[B43] Panagopoulos J. A. Svitra P. P. Puliafito C. A. Gragoudas E. S. (1989). Photodynamic therapy for experimental intraocular melanoma using chloroaluminum sulfonated phthalocyanine. Arch. Ophthalmol-Chic. 107:886. 10.1001/archopht.1989.01070010908039 2730407

[B44] Paris J. L. Cabanas M. V. Manzano M. Vallet-Regi M. (2015). Polymer-grafted mesoporous silica manoparticles as ultrasound-responsive drug carriers. ACS. Nano. 9, 11023–11033. 10.1021/acsnano.5b04378 26456489

[B45] Park C. Oh K. Lee S. C. Kim C. (2007). Controlled release of guest molecules from mesoporous silica particles based on a pH-responsive polypseudorotaxane motif. Angew. Chem. Int. Edit. 46, 1455–1457. 10.1002/anie.200603404 17221893

[B46] Perche F. Biswas S. Wang T. Zhu L. Torchilin V. P. (2014). Hypoxia-targeted siRNA delivery. Angew. Chem. Int. Edit. 53, 3362–3366. 10.1002/anie.201308368 24554550 PMC4150469

[B47] Pervaiz S. Olivo M. (2006). Art and science of photodynamic therapy. Clin. Exp. P. 33, 551–556. 10.1111/j.1440-1681.2006.04406.x 16700893

[B48] Ramamonjisoa N. Ackerstaff E. (2017). Characterization of the tumor microenvironment and Tumor-Stroma interaction by non-invasive preclinical imaging. Front Oncol. 7:3. 10.3389/fonc.2017.00003 28197395 PMC5281579

[B49] Raza A. Hayat U. Rasheed T. Bilal M. Iqbal H. M. N. (2018). Redox-responsive nano-carriers as tumor-targeted drug delivery systems. Eur. J. Med. Chem. 157, 705–715. 10.1016/j.ejmech.2018.08.034 30138802

[B50] Rkein A. M. Ozog D. M. (2014). Photodynamic Therapy. Dermatol. Clin. 32, 415–425. 10.1016/j.det.2014.03.009 24891062

[B51] Roco M. C. (2003a). Converging science and technology at the nanoscale: opportunities for education and training. Nat. Biotechnol. 21, 1247–1249. 10.1038/nbt1003-1247 14520410

[B52] Roco M. C. (2003b). Nanotechnology: convergence with modern biology and medicine. Curr. Opin. Biotech. 14, 337–346. 10.1016/S0958-1669(03)00068-5 12849790

[B53] Shi H. Sun W. Liu C. Gu G. Ma B. Si W.. (2016). Tumor-targeting, enzyme-activated nanoparticles for simultaneous cancer diagnosis and photodynamic therapy. J. Mater. Chem. B. 4, 113–120. 10.1039/C5TB02041G 32262814

[B54] Sun Y. Liang Y. Dai W. He B. Zhang H. Wang X.. (2019). Peptide-drug conjugate-based nanocombination actualizes breast cancer treatment by maytansinoid and photothermia with the assistance of fluorescent and photoacoustic images. Nano. Lett. 19, 3229–3237. 10.1021/acs.nanolett.9b00770 30957499

[B55] Tian K. Jia X. Zhao X. Liu P. (2017). Biocompatible reduction and pH dual-responsive core cross-linked micelles based on multifunctional amphiphilic linear-hyperbranched copolymer for controlled anticancer drug delivery. Mol. Pharm. 14, 799–807. 10.1021/acs.molpharmaceut.6b01051 28186770

[B56] Toole B. P. (1990). Hyaluronan and its binding proteins, the hyaladherins. Curr. Opin. Cell. Biol. 2, 839–844. 10.1016/0955-0674(90)90081-O 1707285

[B57] Upreti M. Jyoti A. Sethi P. (2013). Tumor microenvironment and nanotherapeutics. Transl. Cancer. Res. 2, 309–319. 10.3978/j.issn.2218-676X.2013.08.11 24634853 PMC3951160

[B58] Vordermark D. (2010). Hypoxia-specific targets in cancer therapy: role of splice variants. BMC. Med. 8:45. 10.1186/1741-7015-8-45 20624302 PMC2909930

[B59] Wang H. Han X. Dong Z. Xu J. Wang J. Liu Z. (2019). Hyaluronidase with pH-responsive dextran modification as an adjuvant nanomedicine for enhanced photodynamic-immunotherapy of cancer. Adv. Funct. Mater. 29:1902440 10.1002/adfm.201902440

[B60] Wang Y. Wei G. Zhang X. Xu F. Xiong X. Zhou S. (2017). A step-by-step multiple stimuli-responsive nanoplatform for enhancing combined chemo-photodynamic therapy. Adv. Mater. 29:1605357. 10.1002/adma.201605357 28128876

[B61] Wei H. Zhuo R. Zhang X. (2013). Design and development of polymeric micelles with cleavable links for intracellular drug delivery. Prog. Polym. Sci. 38, 503–535. 10.1016/j.progpolymsci.2012.07.002

[B62] Yang G. Phua S. Z. F. Lim W. Q. Zhang R. Feng L. Liu G.. (2019). A hypoxia-responsive albumin-based nanosystem for deep tumor penetration and excellent therapeutic efficacy. Adv. Mate. 31:1901513. 10.1002/adma.201901513 31069885

[B63] Yang W. Zhang F. Deng H. Lin L. Wang S. Kang F.. (2020). Smart nanovesicle-mediated immunogenic cell death through tumor microenvironment modulation for effective photodynamic immunotherapy. ACS. Nano. 14, 620–631. 10.1021/acsnano.9b07212 31877023

[B64] Yang X. D. Zhu R. Yin J. P. Ma S. Cui J. W. Zhang J. (2019). Synergy of electron transfer and charge transfer in the control of photodynamic behavior of coordination polymers. Chem–Eur. J. 25, 13152–13156. 10.1002/chem.201902300 31350807

[B65] Yang Y. Zhang Y. Wu Q. Cui X. Lin Z. Liu S.. (2014). Clinical implications of high NQO1 expression in breast cancers. J. Exp. Clin. Cancer. Res. 33:14. 10.1186/1756-9966-33-14 24499631 PMC3944477

[B66] Yang Z. Chen Q. Chen J. Dong Z. Zhang R. Liu J.. (2018). Tumor-pH-responsive dissociable albumin–tamoxifen nanocomplexes enabling efficient tumor penetration and hypoxia relief for enhanced cancer photodynamic therapy. Small 14:e1803262. 10.1002/smll.201803262 30307701

[B67] Yao C. Li Y. Wang Z. Song C. Hu X. Liu S. (2020). Cytosolic NQO1 enzyme-activated near-infrared fluorescence imaging and photodynamic therapy with polymeric vesicles. ACS. Nano. 14, 1919–1935. 10.1021/acsnano.9b08285 31935063

[B68] Zhang D. Zheng A. Li J. Wu M. Cai Z. Wu L.. (2017). Tumor microenvironment activable self-assembled DNA hybrids for pH and redox dual-responsive chemotherapy/PDT treatment of hepatocellular carcinoma. Adv. Sci. 4:1600460. 10.1002/advs.201600460 28435778 PMC5396159

[B69] Zhang N. Zhao F. Zou Q. Li Y. Ma G. Yan X. (2016). Multitriggered tumor-responsive drug delivery vehicles based on protein and polypeptide coassembly for enhanced photodynamic tumor ablation. Small 12, 5936–5943. 10.1002/smll.201602339 27622681

[B70] Zhu H. Fang Y. Miao Q. Qi X. Ding D. Chen P.. (2017). Regulating near-infrared photodynamic properties of semiconducting polymer nanotheranostics for optimized cancer therapy. ACS. Nano. 11, 8998–9009. 10.1021/acsnano.7b03507 28841279

[B71] Zhu R. He H. Liu Y. Cao D. Yan J. Duan S.. (2019). Cancer-selective bioreductive chemotherapy mediated by dual hypoxia-responsive nanomedicine upon photodynamic therapy-induced hypoxia aggravation. Biomacromolecules 20, 2649–2656. 10.1021/acs.biomac.9b00428 31125209

